# Updated taxonomy of LactifluussectionLuteoli: *L.russulisporus* from Australia and *L.caliendrifer* from Thailand

**DOI:** 10.3897/mycokeys.56.35204

**Published:** 2019-07-10

**Authors:** Glen Dierickx, Marie Froyen, Roy Halling, Komsit Wisitrassameewong, Lynn Delgat, Eske De Crop, Annemieke Verbeken

**Affiliations:** 1 Research Group Mycology, Department of Biology, Ghent University, Ghent, Belgium Ghent University Ghent Belgium; 2 Institute of Systematic Botany, The New York Botanical Garden, Bronx, NY, USA The New York Botanical Garden New York United States of America; 3 National Center for Genetic Engineering and Biotechnology (BIOTEC), Chang Wat Pathum Thani, Thailand BIOTEC Thailand Science Park Thailand

**Keywords:** Ectomycorrhizal fungi, *Russulaceae*, milkcaps, taxonomy, phylogeny, *Leptocystidia*, sterile elements, paracystidia

## Abstract

*Lactifluusrussulisporus* Dierickx & De Crop and *Lactifluuscaliendrifer* Froyen & De Crop are described from eucalypt forests in Queensland, Australia and different forest types in Thailand, respectively. Both species have recently been published on Index Fungorum and fit morphologically and molecularly in L.sect.Luteoli, a section within L.subg.Gymnocarpi that encompasses species with alboochraceous basidiomes, white latex that stains brown and typical capitate elements in the pileipellis and/or marginal cells.

## Introduction

Since the division of *Lactarius* into *Lactarius* sensu novo and *Lactifluus* (Buyck et al. 2008), our understanding of both genera has increased significantly. Although *Lactifluus* is the smaller of the two genera, it is characterised by a higher genetic diversity with subgroups in very different and genetically distant clades (De Crop et al. 2017). Recently, efforts in *Lactifluus* culminated in a new infrageneric classification based on a multi-gene phylogeny (De Crop et al. 2017). Herein, the genus *Lactifluus* is subdivided into four subgenera: L.subg.Lactariopsis, L.subg.Lactifluus, L.subg.Pseudogymnocarpi and L.subg.Gymnocarpi. The latter contains four sections, apart from five isolated species and one unnamed clade: L.sect.Gymnocarpi and L.sect.Phlebonemi with exclusively African representatives, L.sect.Tomentosi with representatives from Oceania and L.sect.Luteoli with seven species spanning all continents, except South America. De Crop et al. (2017) illustrates the existence of two new sister species, one from Thailand and one from Australia, within the latter section. These two sister species were recently published on Index Fungorum (Dierickx et al. 2019) with a short description, but are fully described in this paper: *L.caliendrifer* from Thailand and *L.russulisporus* from Australia. While in De Crop et al. (2017) four loci (ITS, LSU, *RPB1* and *RPB2*) were used to construct the phylogeny, here only ITS is used.

The Thai collections were found in different habitats: KW 378 was found in montane forest with Fagaceae trees (*Lithocarpus*, *Castanopsis* and *Quercus*) and some bamboo tree species; KW 392 was growing in disturbed Dipterocarp forest, with *Dipterocarpus* spp. The first Australian collection, RH 9398, was growing on sand in wet sclerophyll forest, in the presence of various Myrtaceae (*Leptospermum*, *Syncarpia*, *Eucalyptuspilularis* and *E.microcorys*). It is a closed canopy forest but receives less rainfall than ‘true’ rainforest. The second collection, RH 9674, was found in subtropical rainforest; nearby vegetation includes *Eucalyptus* spp. and *Lophostemon* spp. (Myrtaceae).

## Methods

### Sampling

The two collections of *Lactifluuscaliendrifer* were made during fieldwork by Komsit Wissitrassameewong in 2012 and are deposited in Herbarium Universitatis Gandavensis, Belgium (GENT) and the herbarium of Mae Fah Luang University, Chiang, Thailand (MFLU). For *L.russulisporus*, fieldwork in 2010 and 2012 by Roy Halling and collaborators resulted in two collections of the species, which are deposited in The William and Lynda Steere Herbarium of the New York Botanical Garden (NY) and the Queensland Herbarium (BRI). We know from earlier research (De Crop et al. 2017; De Crop et al. 2016) that Halling 9398 and Wisitrassameewong 378 belong to L.subg.Gymnocarpisect.L.teoli. Our dataset contains the ITS sequences used for L.subg.Gymnocarpi in De Crop et al. (2017), supplemented with newly generated sequences. Five species of L.subg.Lactifluus were used as outgroup.

### Morphology

Macroscopic characters are all based on fresh material. Microscopic features were studied from dried material in Congo red in SDS. Possible excretory products were checked for in Cotton blue in lactic acid and Cresyl blue (Clémençon 1997; 2009) Spore ornamentation is described and illustrated as observed in Melzer’s reagent. A total of 40 spores (20 per collection) were measured for each of the two new species. For details on terminology we refer to Verbeken (1998) and Verbeken and Walleyn (2010). Line-drawings were made with the aid of a drawing tube (Zeiss camera lucida on a Zeiss Axioskop 2 microscope equipped with a magnification changer of 2.5× for spores and an Olympus U-DA on an Olympus CX21 microscope for individual elements and pileipellis structures) at original magnifications: 6000× for spores, 1500× for individual elements and sections. Basidia length excludes sterigmata length. Spores were measured in side view, excluding the ornamentation, and measurements are given as (MINa) [AVa-2*SD]–AVa–AVb–[AVb+2*SD] (MAXb), with AVa = lowest mean value for the measured collections and AVb = greatest mean value for the measured collections, SD = standard deviation, MINa = lowest extreme value of collection “a” and MAXb = greatest extreme value of collection “b”. The Q-value (quotient length/width) is given as (MIN Qa) Qa–Qb (MAX Qb), with Qa = lowest mean ratio for the measured collections and Qb = greatest mean ratio for the measured collections, MIN Qa = lowest extreme ratio of collection “a” and MAX Qb = greatest extreme ratio of collection “b”. Other measurements are given as MIN–MAX values. Colour codes refer to Kornerup and Wanscher (1978). Microscopic photographs were taken using a Nikon eclipse NI-U–microscope equipped with a DX-Fi1c camera and Nikon NIS-Elements software including EDF module.

### Molecular work

DNA from dried collections was extracted using the protocol described by Nuytinck and Verbeken (2003) with modifications described in Van de Putte et al. (2010), and from fresh material using the CTAB extraction method described in Nuytinck and Verbeken (2003). Protocols for PCR amplification follow Le et al. (2007). The internal transcribed spacer (ITS) was sequenced for a second collection for each new species using the primers ITS1-F and ITS4 (Gardes and Bruns 1993; White et al. 1990). PCR products were sequenced using an automated ABI 3730 XL capillary sequencer (Life Technology) at Macrogen. Forward and reverse sequences were assembled into contigs and edited where needed with SequencherTM v5.0 software (Gene Codes Corporation, Ann Arbor, MI, USA).

### Phylogenetic analysis

Sequences were aligned online using the E-INS-I strategy of the multiple sequence alignment program MAFFT v7 (Katoh and Standley 2013). Trailing ends were trimmed, and where necessary, the alignment was manually edited in MEGA 7 (Kumar et al. 2016). The alignment can be obtained from the first author and TreeBASE (Submission ID S23999). The best partition scheme was selected with PARTITIONFINDER 2 (Lanfear et al. 2016) using standard settings. Aligned sequences were partitioned into 18S (1–56), ITS1 (57–334), 5.8S (335–482), ITS2 (483–820) and 28S (821–868). Maximum likelihood (ML) analyses were conducted with RAxML v8.2.10 (Stamatakis 2014), where a ML analysis was combined with the Rapid Bootstrapping algorithm with 1000 replicates under the GTRCAT option (Stamatakis et al. 2008). All analyses were performed on the CIPRES Science Gateway (Miller et al. 2015).

## Results

In congruence with De Crop et al. (2017), our molecular results show that the collections from Australia as well as those from Thailand belong to Lactifluus.subg.Gymnocarpisect.Luteoli (Fig. 2). The newly generated sequences for Halling 9674 and Wisitrassameewong 392 belong to the same species as Halling 9398 and Wisitrassameewong 378 respectively. These two species are supported by morphological and geographical differences (see discussion) and are fully described below as *L.russulisporus* and *L.caliendrifer*.

### Taxonomy

#### Lactifluus russulisporus

Taxon classificationFungiRussulalesRussulacea

Dierickx & De Crop

829913

Index Fungorum 392: IF 829913

Figs 1 , 3 , 4

##### Original diagnosis.

Basidiocarps small (up to 4 cm cap diam.). Cap and stipe dry, matt, yellowish white to pale brown. Context with unpleasant, fishy smell. Latex copious, watery white, staining tissues brown. Basidiospores broadly ellipsoid 7.0–*7.8–7.9*–8.7 × 5.7–*6.4–6.5*–7 μm (n=40, Q = 1.14–*1.23*–1.40); ornamented with irregular and isolated warts which are up to 1.3 μm high. True pleurocystidia absent, but with few to abundant sterile elements in the hymenium. Pileipellis a lampropalisade. *L.russulisporus* differs from its sister species, *L.caliendrifer*, by its longer basidia, slightly bigger spores with a somewhat heavier and more irregular ornamentation and the absence of abundant thick-walled marginal cells.

**Figure 1.**
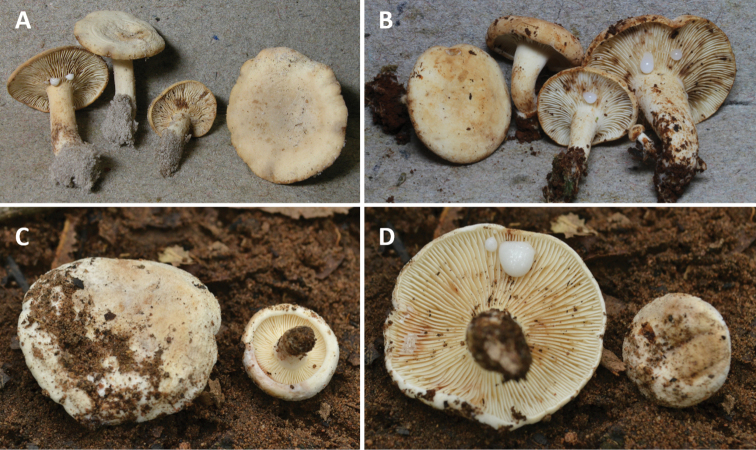
**A–B***Lactifluusrussulisporus* basidiomes **C–D***L.caliendrifer* basidiomes **A** holotype, RH 9398 **B** RH 9674 **C** holotype, KW 378 **D** KW 392.

**Basidiomes** rather small. **Pileus** 20–40 mm diam., convex to plano-convex and depressed on disc to uplifted and slightly depressed, yellowish white (4A2) to pale brown, dry, matted, subtomentose to finely subvelutinous and somewhat subrugulose to subcorrugate; margin inrolled. **Stipe** 10–30 × 5–10 mm cylindrical, dry, matt, yellowish white, sometimes paler brownish towards the base, with white mycelium at the base. **Lamellae** adnexed to subdecurrent, rather close, pale greyish white to yellowish white, turning darker to near pale brown with age. **Context** white, solid to somewhat pithy in the stipe; smell unpleasant, fishy; taste mild. **Latex** copious, watery white, staining tissues brown.

**Basidiospores** broadly ellipsoid 7.0–*7.8–7*,*9*–8.7 × 5.7–*6.4–6*,*5*–7 µm (n=40, Q = 1.14–*1.23*–1.40); ornamentation amyloid, prominent, composed of irregular and isolated warts which are up to 1.3 µm high, never forming a reticulum; plage distinct and inamyloid. **Basidia** 43–71 × 8–14 µm, subcylindrical to subclavate, thin-walled, mostly 4-spored. **Pleurolamprocystidia** absent. **Sterile elements** inconspicuous to abundant, cylindrical, sometimes a bit irregular, 17–64 × 3–7 µm, thin-walled and up to 3-septate, sometimes emerging, with terminal cells 9–39 × 2.5–6.5 µm. **Pleuropseudocystidia** generally abundant, sometimes emerging, 3–8 µm diam., irregularly cylindrical; apex obtuse to subcapitate; content oil-like to granular. **Lamellae edge** sterile, marginal cells 23–74 × 2–7.5 µm, thin-walled, cylindrical to subfusiform or slightly subclavate, often branched, not septate or with up to 3 septae, with terminal cells 7–49 × 2–7.5; apex obtuse to subcapitate; some marginal cells may be slightly thick-walled, but these are scarce. **Hymenophoral trama** cellular, with lactifers. **Pileipellis** a lampropalisade; elements of the suprapellis 35–180 × 2.5–6 µm, cylindrical, thick-walled and often septate; apex obtuse to capitate; subpellis cellular, composed of isodiametric, sometimes slightly thick-walled cells, which are 7–30 µm diam. **Stipitipellis** a trichoderm to lamprotrichoderm; ascending hyphae 35–80 × 4–6 µm, up to 3 septate, slightly thick-walled to thick-walled especially basal cells, apex obtuse to capitate. **Clamp connections** absent.

##### Distribution.

Known from Eastern Australia.

##### Ecology.

East-Australian wet sclerophyll and subtropical rainforest, scattered to gregarious on soil under *Leptospermum*, *Syncarpia*, and *Eucalyptus* spp.

##### Etymology.

Named after the spores which are reminiscent of the spore ornamentation and shape of many *Russula* species.

##### Conservation status.

Unknown.

##### Specimens examined.

Australia. Queensland West of Brisbane, D’Aguilar National Park, Maiala Area walking tracks, alt. 680 m, 27°20'0.3"S, 152°45'48.3"E, rainforest, scattered on the soil near *Eucalyptus* sp. and *Lophostemon* sp., 8 March 2012, R. E. Halling and N. Fechner, R.E.H. 9674 (BRI, NY); Queensland: Fraser Island, Wanggoolba Creek Road, West of Central Station, alt. 90m, 25°28'S, 153°2'E, gregarious on sand with *Leptospermum*, *Syncarpia*, *Eucalyptuspilularis* and *Eucalyptusmicrocorys*, 27 May 2010, leg.: R. E. Halling, N. Fechner and M. Castellano, R.E.H. 9398 (holotypus BRI, isotypus NY).

**Figure 2.**
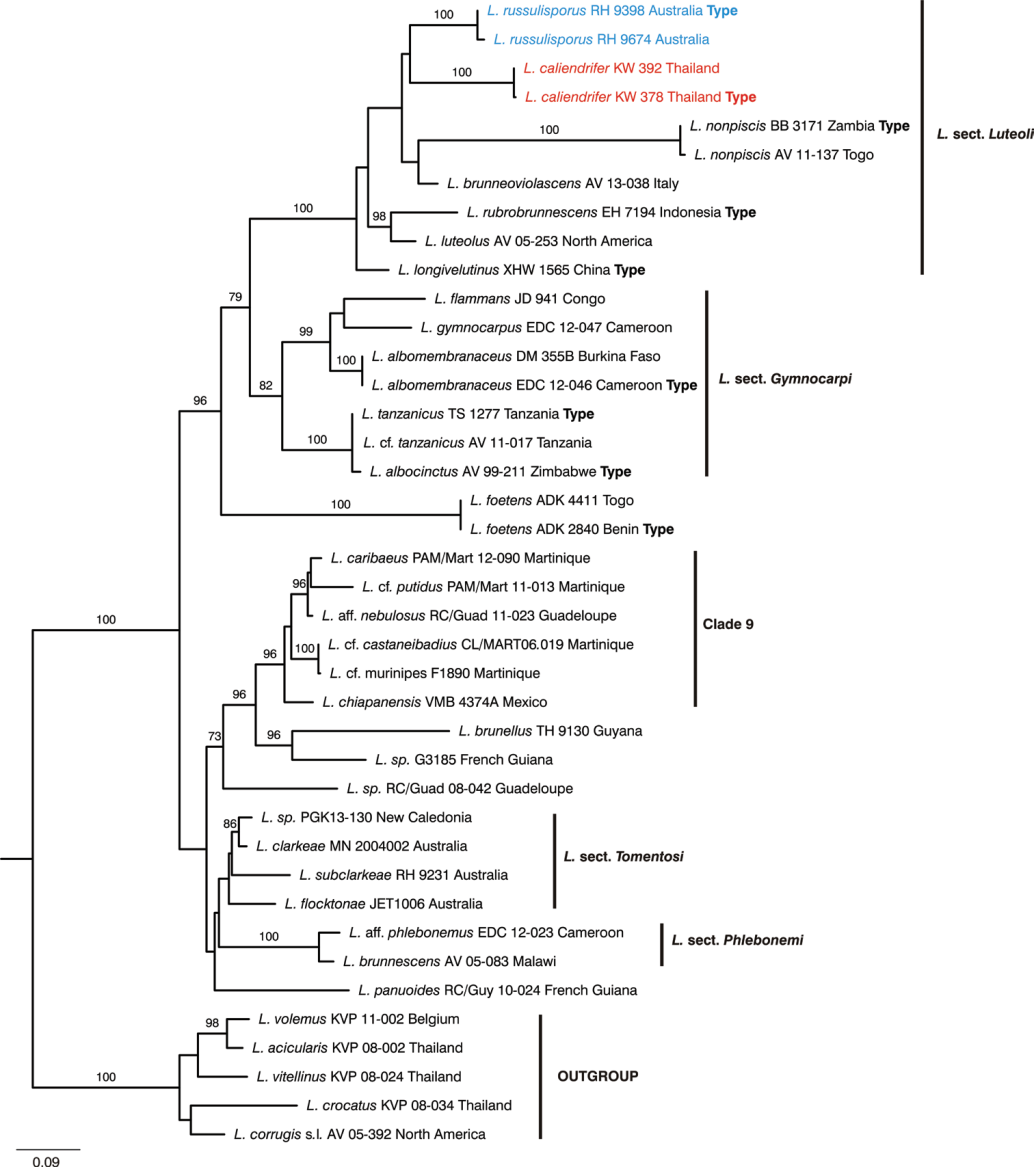
Overview Maximum Likelihood tree of Lactifluussubg.Gymnocarpi, based on ITS sequence data. Maximum Likelihood bootstrap values >70 are shown.

**Figure 3.**
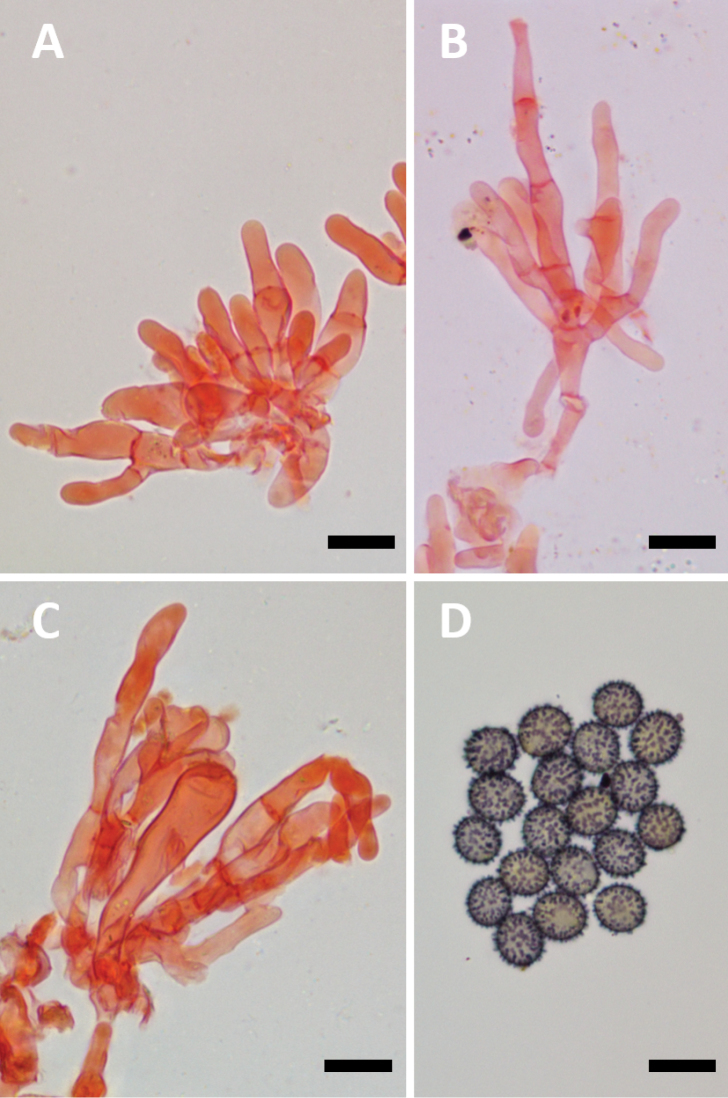
**A–D** Microscopic characters of *Lactifluusrussulisporus***A** marginal cells, RH 9764 **B** marginal cells, holotype, RH 9398 **C** basidiole and sterile elements, holotype, RH 9398 **D** spores, holotype, RH 9398. Scale bar: 10 µm.

**Figure 4.**
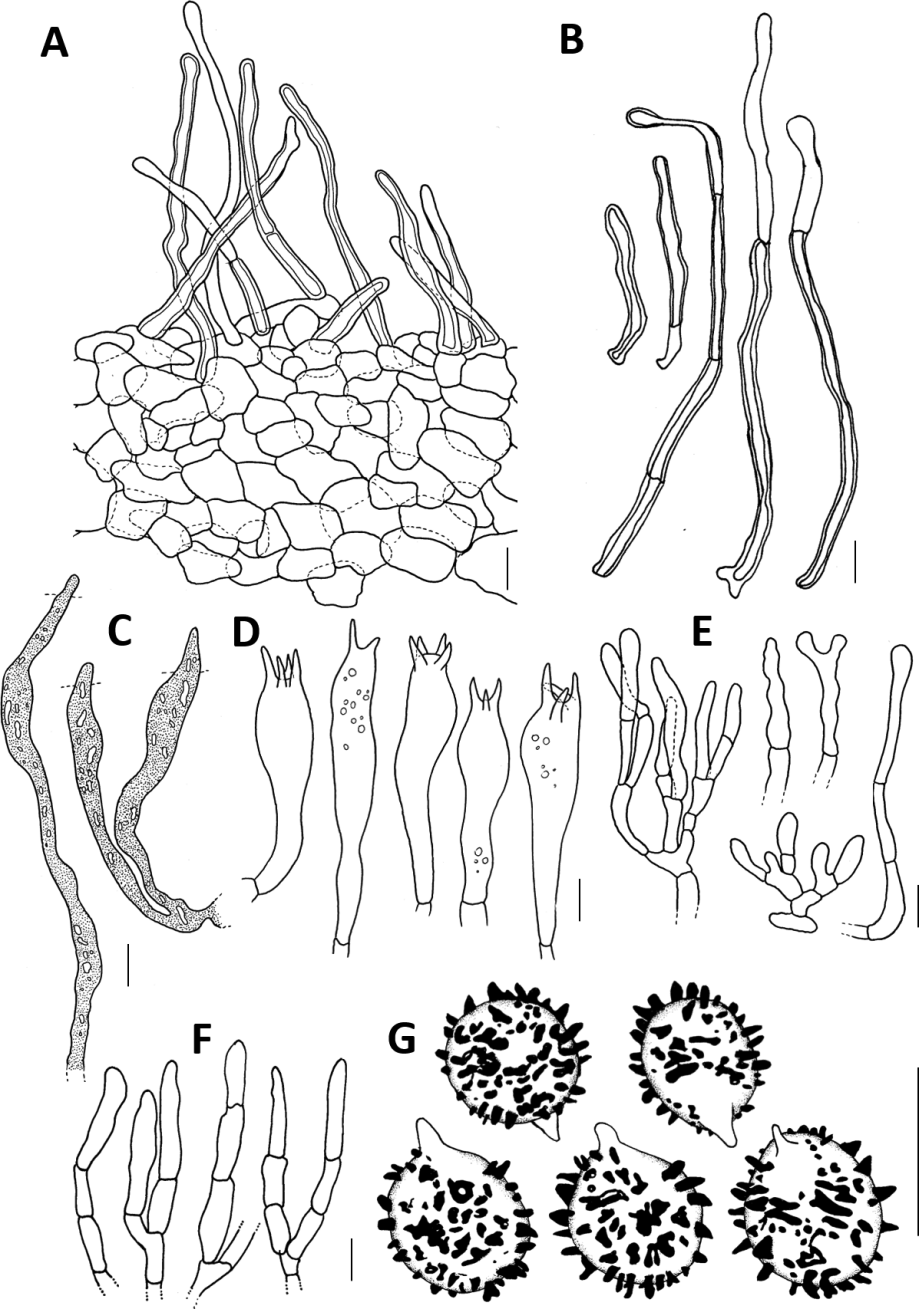
Microscopic features of *Lactifluusrussulisporus***A** section through the pileipellis **B** pileipellis hairs **C** pseudocystidia **D** basidia **E** marginal cells **F** sterile elements from the hymenium **G** basidiospores. Illustrations by G. Dierickx and A. Verbeken. Scale bars: 10 µm.

##### Remarks.

*Lactifluusrussulisporus* differs from its sister species, *L.caliendrifer*, by its longer basidia, slightly bigger spores with a somewhat heavier and more irregular ornamentation and the absence of abundant thick-walled marginal cells.

#### Lactifluus caliendrifer

Taxon classificationFungiRussulalesRussulacea

Froyen & De Crop

829914

Figs 1 , 5 , 6

##### Original diagnosis.

Basidiocarps small (up to 3.5 cm cap diam.) and turning brown when bruised. Cap very velvety to tomentose, white to cream-coloured. Stipe smooth to velvety, white. Context with smell fruity, strong. Latex copious, watery white to white, sticky, turning dark yellow to mustard brown; taste acrid and a bit bitter. Basidiospores broadly ellipsoid, (5.8) 5.9–*7.0–7.1*–7.9 × (4.5) 4.7–*5.6–5.7*–6.2 μm (n=40, Q = 1.12–*1.24*–1.41); ornamented with irregular and isolated warts which are up to 1 μm high. True pleurocystidia absent, but with sterile elements in the hymenium. Pileipellis a palisade to lampropalisade. *L.caliendrifer* differs from its sister species, *L.russulisporus*, by the abundant thick-walled marginal cells, very long pileipellis hairs and slightly smaller basidia and spores with more regular and lower warts.

**Basidiomes** rather small. **Pileus** 19–34 mm diam., planoconvex, sometimes centrally depressed; surface very velvety, dull, pruinose, tomentose, covered with hairs in tufts, white to cream-coloured, becoming brown after bruising; margin inflexed. **Stipe** 11–17 × 4–7 mm, cylindrical, centrally attached; surface smooth to velvety, white, turning brownish when bruised. **Lamellae** adnate to decurrent, narrow and thin, 0.5–1.5 mm broad, crowded, with 3 to 4 lamellulae of different lengths between 2 lamellae, whitish, concolorous with pileus and becoming brownish when bruised; edge entire, concolorous. **Context** white, changing to pale pinkish near pileipellis after a while, turning brown when broken (6E8) or sometimes paler caramel (6C6), or camel (6D4); smell fruity, strong; taste unknown. **Latex** copious, watery white to white, sticky, turning dark yellow (4C8) after a few minutes, later mustard brown (5E6) after 15 minutes; taste acrid and a bit bitter.

**Basidiospores** broadly ellipsoid, (5.8) 5.9–*7.0–7.1*–7.9 × (4.5) 4.7–*5.6–5.7*–6.2 µm (n=40, Q = 1.12–*1.24*–1.41); ornamentation amyloid, composed of irregular or isolated warts which are up to 1 µm high, sometimes connected by low ridges, but not forming a reticulum; plage inamyloid. **Basidia** 27–55 × 8–12 µm, subcylindrical to subclavate, thin-walled, mostly 4-spored; content oil-like to granular. **Pleurolamprocystidia** absent. **Sterile elements** cylindrical, 28–52 × 4–8 µm, thin-walled and up to 3-septate, slightly emerging, with terminal cells 6–28 × 4–7.5 µm. **Pleuropseudocystidia** rare to abundant, 4–10 µm diam., emerging, irregularly cylindrical; apex obtuse to subcapitate; content oil-like to granular. **Lamellae edge **sterile. **Marginal cells** 28–61 × 3–6 µm, often septate: with 1 to 5 septae, with terminal cells up to 47 µm long, thick-walled, occasionally branched; apex obtuse to subcapitate. **Hymenophoral trama** cellular, with lactifers. **Pileipellis** a palisade to lampropalisade, elements of the suprapellis 60–440 × 2.5–5 µm; cylindrical, septate, sometimes capitate, slightly thick-walled; subpellis composed of isodiametric, mostly thin-walled cells. **Stipitipellis** a trichoderm to lamprotrichoderm; ascending hyphae 10–75 × 3–6 µm, up to 2 septate, often thick-walled, apex obtuse to capitate. **Clamp connections** absent.

##### Distribution.

Known from Thailand.

##### Ecology.

Thai montane and dipterocarp forest, growing under *Dipterocarpus*, *Lithocarpus*, *Castanopsis* and *Quercus*.

##### Etymology.

Means ‘wearing a wig’, referring to the long hairs in the pileipellis.

##### Conservation status.

Unknown.

##### Additional material examined.

Thailand. Thoeng district, Chiang Rai, alt. 420 m, 19°36'45"N, 100°04'00"E, Forest roadside, dry dipterocarp forest (Longan plantation), 20 August 2012, K. Jatuwong, Wisitrassameewong 392 (GENT, MFLU); Doi Pui, Chiang Rai, alt. 650 m, 19°49'26"N, 99°52'19"E, bamboo forest, 3 July 2012, leg.: Wisitrassameewong 378 (holotypus, GENT, isotypus MFLU).

**Figure 5.**
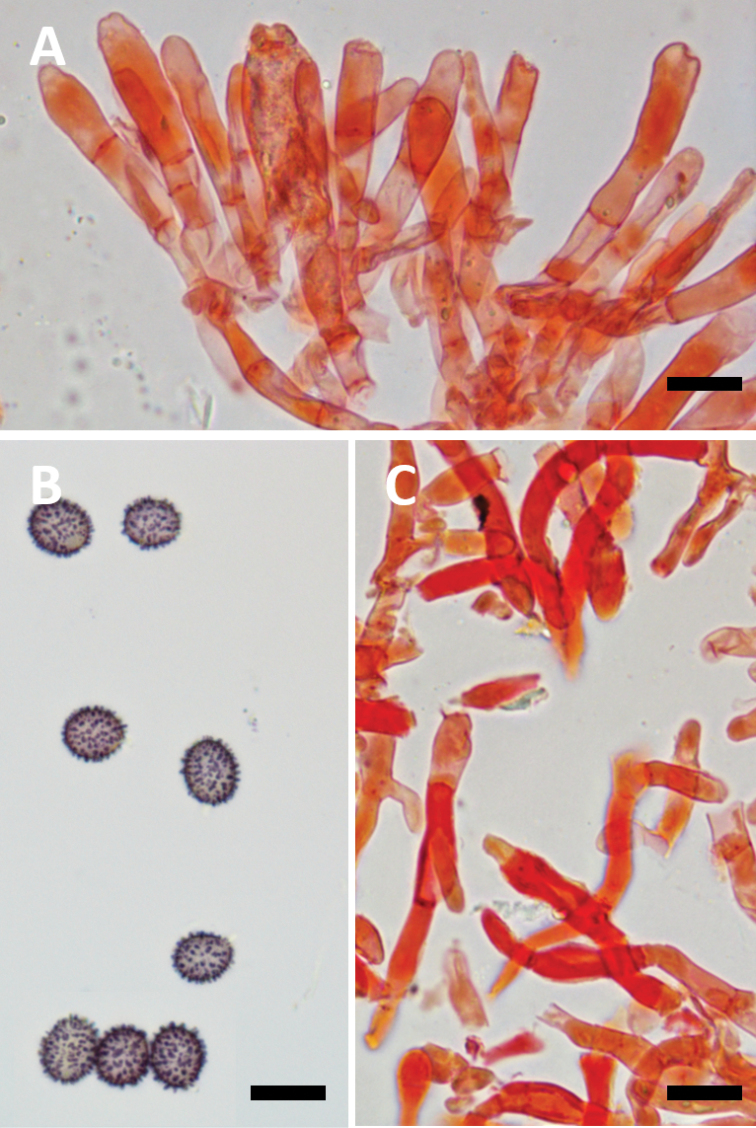
**A–C** Microscopic characters of *Lactifluuscaliendrifer***A** basidiole and sterile elements, KW 392 **B** spores, holotype, KW 378 **C** marginal cells, holotype, KW 378. Scale bar: 10 µm.

**Figure 6.**
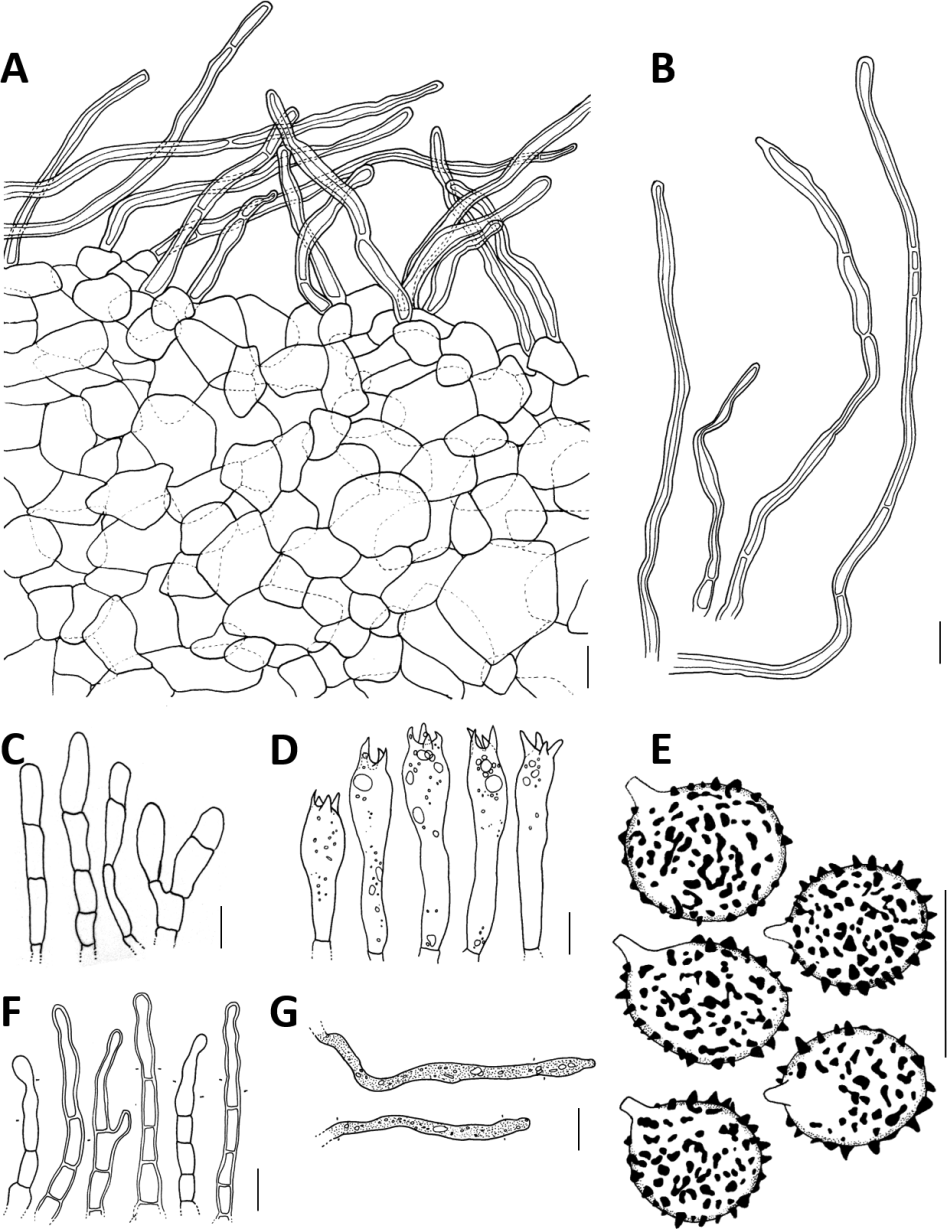
Microscopic features of *Lactifluuscaliendrifer*. **A** section through the pileipellis **B** pileipellis hairs **C** sterile elements from the hymenium **D** basidia **E** basidiospores **F** marginal cells **G** pseudocystidia. Illustrations by M. Froyen, G. Dierickx and A. Verbeken. Scale bar: 10 µm.

##### Remarks.

*Lactifluuscaliendrifer* differs from its sister species, *L.russulisporus*, by the abundant thick-walled marginal cells, very long pileipellis hairs and slightly smaller basidia and spores with more regular and lower warts.

## Discussion

The morphological distinction between *Lactarius* and *Lactifluus* is not always straightforward in the field and can only be based on some general trends. For example, the genus *Lactifluus* is generally characterised by the complete absence of zonate and viscose to glutinose caps, and it contains many species with veiled and velvety caps (Buyck et al. 2008; De Crop et al. 2017; Verbeken and Nuytinck 2013). A cellular hymenophoral trama and a lampropalisade as pileipellis structure are both characters which are more often observed in *Lactifluus* than in *Lactarius*.

The newly described species can macroscopically be recognised as members of genus *Lactifluus* by the tomentose to velvety appearance of their caps and the exuded milk that changes to brownish (which is more common in *Lactifluus* and very rare in *Lactarius*). Microscopically the presence of a lampropalisade and a cellular trama indicate the affinity with *Lactifluus*.

*Lactifluusrussulisporus* and *L.caliendrifer* belong to L.subg.Gymnocarpi, which is supported by molecular (Fig. 2) (De Crop et al. 2017) and morphological data (e.g. brown discolouration of the latex and the absence of true pleurolamprocystidia). Both new species are placed in L.sect.Luteoli, which consists of seven species from all continents except South America and Antarctica, and are characterised by capitate elements in the pileipellis and/or the presence of differentiated marginal cells.

The sister species *Lactifluusrussulisporus* and *L.caliendrifer* are clearly delimited molecularly, which is reflected in both geographical and morphological characters. Geographically, *L.russulisporus* is only known from Eastern Australia (Queensland), while *L.caliendrifer* is only known from Southeast Asia (Thailand). In the field, both species can be recognised by their cream to yellowish white basidiomes, dry and finely velvety to pruinose pilei, rather crowded white to concolorous lamellae and copious watery latex that stains brown. These features are common to most species in L.sect.Luteoli.

*Lactifluuscaliendrifer* can be distinguished macroscopically by its velvety pileus, whiter basidiomes and its strong and fruity smell. *Lactifluusrussulisporus* differs from its sister species by having a more yellowish-brown shade and an unpleasant, fishy smell.

Microscopically, the two species can be differentiated by several characters. First, the pileipellis elements are (35) 85–125 (180) µm long in *Lactifluusrussulisporus*, while they can exceed 400 µm in *L.caliendrifer*. Second, *L.russulisporus* has larger spores: on average 7,8–7.9 × 6.3–6.4 µm (*L.russulisporus*) versus 7.0–7.1 × 5.6–5.7 µm (*L.caliendrifer*), which is reflected in basidia size: 43–71 × 8–14 µm vs. 27–55 × 8–12 µm for *L.russulisporus* and *L.caliendrifer* respectively. Third, *L.caliendrifer* is characterised by the presence of numerous thick-walled marginal cells, while these are scarce and therefore difficult to find in *L.russulisporus*. Lastly, the ascending hyphae of the stipitipellis are often shorter in *L.caliendrifer*: 10–75 µm versus 35–80 µm long for *L.caliendrifer* and *L.russulisporus* respectively.

Five other species occur in Lactifluussect.Luteoli. *Lactifluuslongivelutinus* is known from China and differs from both new species by its often eccentrical to almost lateral stipe, marginal cells with globose apex containing brownish content, and long, thick-walled terminal cells of the stipitipellis (80–150 (200) µm) (Wang and Verbeken 2006). Comparable to *L.caliendrifer*, it possesses long pileipellis elements (300–400 × 3.5–5 (6.0) µm).

*Lactifluusrubrobrunnescens* is known to occur in Java (Indonesia) and can easily be recognised by a hollow stipe, latex that stains reddish brown, more globose spores (average Q = 1.16) and distinctly capitate elements in the pilei- and stipitipellis, and marginal cells (Verbeken et al. 2001).

*Lactifluusnonpiscis* has an African distribution and is well characterised by the purplish brown staining basidiomes with a strongly wrinkled to rugulose pileus. In addition, *L.nonpiscis* can be discerned by the shorter elements of the suprapellis (40–80 (100) µm) and the slightly larger and more ellipsoid spores (8–*8.7–9.2*–10.0 × 6.1–*6.6–6.7*–7.3 µm, Q = 1.21–*1.31–1.36*–1.49) (Verbeken and Walleyn 2010). *Lactifluusbrunneoviolascens* and *L.luteolus* are two look-a-likes, the first one in Europe, the second one in North America. They differ from the other representatives by their larger basidiome size (pileus 50–80 mm, stipe 40–70 × 10–12 mm). *Lactifluusluteolus* further differs from the two species described here by its more ellipsoid spores (7–8.5 × 5.5–6 µm) that bear slightly lower ornamentation (up to 0.8 µm) and shorter pileipellis hairs (34–70 × 3–5 µm). *Lactifluusbrunneoviolascens* is characterised by abundant capitate, slender and sometimes thick-walled marginal cells.

### Notes on terminology

When it comes to terminology used in the genera *Lactarius* and *Lactifluus*, most authors tend to follow Verbeken and Walleyn (2010) and Verbeken (1998). Unfortunately, some confusion seems to exist concerning hymenophoral cells that can be termed either leptocystidia or sterile elements. Even though this type of cell is frequently present in *Lactifluus* (pers. observations), these cells are only rarely reported in species descriptions (De Crop et al. 2019; Delgat et al. 2017), probably often being dismissed as basidioles and/or of limited taxonomic value. This problem presented itself during the description of the two new species and a consensus between the authors of this paper was pursued.

**Figure 7.**
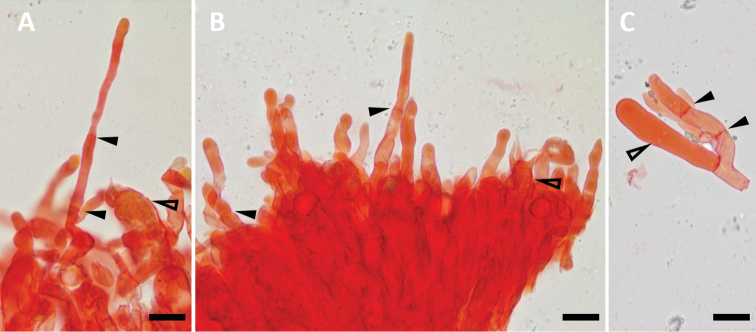
**A–C** Sterile elements of *Lactifluusrussulisporus*, full arrows indicate septa of sterile elements, hollow arrows indicate a basidiole or basidium. **A–B** Protruding sterile cells, holotype, RH 9398 **C** not-protruding sterile element, RH 9674. Scale bar: 10 µm.

The term leptocystidium is composed of the Greek leptós, meaning “smooth, thin-walled” and cystidium, meaning “a sterile body, frequently of distinctive shape, occurring at any surface of a basidiome, particularly the hymenium from which it frequently projects”(Ainsworth 2008). In Clémençon (1997), leptocystidia are described in a similar manner, with the addition that they often have an excretory function. For the latter, we could not find evidence in our collections. According to Verbeken and Walleyn (2010), leptocystidia can be regarded as “thin-walled cystidia without remarkable content and thus only deviating by their shape. They are tapering at the top and often have a rostrate apex, which makes them easy to confuse with monosterigmatic basidia. One can consider them to be cystidia if they are regularly observed and if they never bear a spore or spore primordium”. In the two new species, and by extension in most *Lactifluus* species, thin-walled sterile cells with no remarkable content occur in the hymenium. Furthermore, they do not exhibit a deviating shape, being cylindrical and usually ending blunt. If shape deviation is seen as a vital component for being a cystidium, these cells cannot be named as such. In addition, we dismiss the idea that these cells represent basidioles. Firstly, no intermediate forms between these cells and basidioles were observed. Secondly, in *L.russulisporus* these cells display a different morphology in both collections. In RH 9674, and by extension in general, they do not protrude from the hymenium and do not exhibit a deviant form, leaving open the possibility that they constitute basidioles or protobasidia (Fig. 7C). However, in RH 9398, they grow out strikingly, protruding clearly from the hymenium (Fig. 7A, B). The same behaviour is seen in the pseudocystidia and marginal cells in this collection. According to Moore (2005), principle nine of fungal developmental biology states that “meiocytes appear to be the only hyphal cells that become committed to their developmental fate. Other highly differentiated cells retain totipotency–the ability to generate vegetative hyphal tips that grow out of the differentiated cell to re-establish a vegetative mycelium.” A possible hypothesis is that some stimulus, perhaps environmental, caused the totipotent cells in the hymenium to grow out, giving rise to the protruding sterile elements, pseudocystidia and marginal cells in RH 9398. This explanation adds to the idea that these cells are not precursor cells of meiocytes (basidia).

**Figure 8.**
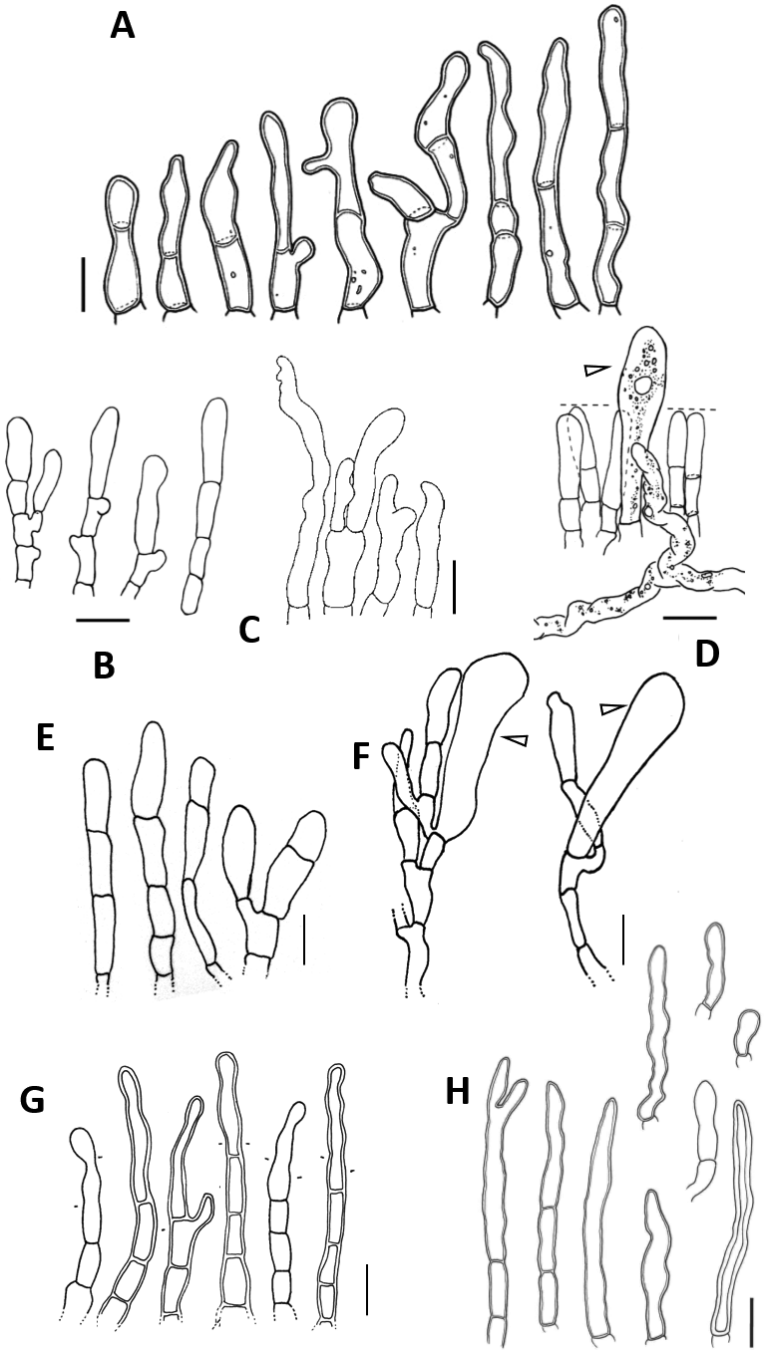
**A–F** Sterile cells in *Lactifluus***G, H** marginal cells in *Lactifluus* with striking resemblance to different sterile cells **A***L.persicinus* from Delgat et al. (2017)**B, D***L.bicapillus* from De Crop et al. (2019)**C** ‘leptocystidia’ from (Verbeken and Walleyn 2010) **E***L.caliendrifer***F***L.russulisporus***G***L.caliendrifer***H***L.albomembranaceus* from (De Crop et al. 2016). Scale bar: 10 µm, arrows indicate basidioles.

As these sterile elements are argued not to be cystidia or basidioles, the question remains as to what they are. Several terms might have been used to indicate the same kind of cells. For example, haplohyphidia refers to unmodified, unbranched or little branched terminal hyphae in the hymenium of (mostly) Aphyllophorales. An intriguing term, paraphyses, is used in the works on the developmental biology of the hymenium done in *Coprinopsiscinerea* (Horner and Moore 1987; Rosin and Moore 1985a). These cells originate as branches of sub-basidial cells and insert into the basidial layer, later inflating so that they become the main structural component as a pavement from which basidia and cystidia protrude (Horner and Moore 1987; Moore 1985; Rosin and Moore 1985a; b). This description fits well with the sterile elements observed in *Lactifluus* (Figs 7, 8F). Nevertheless, paraphyses is a term strongly associated with Ascomycota, used for more hair-like (filiform) cells. It cannot be stated with certainty that Ascomycete paraphyses are homologous to the cells we find in *Lactifluus*.

Given the lack of a distinctive deviating shape in most cases, the improbability of being basidioles and the neutrality of the term, we recommend the use of the term ‘sterile elements’ over the terms ‘leptocystidia’ and ‘paraphyses’ to refer to these cells.

Thereto can be added that marginal cells often bear a striking resemblance to sterile elements (Fig. 8). Furthermore, in *Inocybe*, little differentiated cystidia are referred to as paracystidia, which also show similar morphology to marginal cells and might constitute the same type of cell (Jacobsson and Larsson 2012; Kuyper 1986). Presently it is difficult to argue whether this is due to homology or homoplasy. Marginal cells are sterile elements on a sterile edge that differ from pleurocystidia and are, in fact, ‘hairs’ sensu Romagnesi (Verbeken and Walleyn 2010). In species where the edge is fertile, sterile elements are also present on the edge. It is possible that, when no differentiated marginal cells are present on an infertile edge, sterile elements are present and consequently reported as being marginal cells. We suggest paying more attention to these sterile elements which occur predominantly in *Lactifluus*. Given the variation that we observe within *L.russulisporus*, it is likely that the taxonomic value of this character is rather low, but this needs more observations.

**Table 1.** Specimens and GenBank accession numbers of DNA sequences used in molecular analyses.

Species	Voucher collection (herbarium)	Country	ITS accession no.	Reference
**Lactifluussubg.Gymnocarpi**				
*Lactifluusalbocinctus* **Type**	AV 99-211 (GENT)	Zimbabwe	KR364117	De Crop et al. (2017)
*Lactifluusalbomembranaceus* **Type**	EDC 12-046 (GENT)	Cameroon	KR364064	De Crop et al. (2017)
*Lactifluusalbomembranaceus*	DM 355B	Burkina Faso	LN651269	Maba et al. (2015)
*Lactifluusbrunellus*	TH 9130 (BRG, DUKE)	Guyana	JN168728	Smith et al. (2011)
*Lactifluusbrunneoviolascens*	AV 13-038 (GENT)	Italy	KR364123	De Crop et al. (2017)
*Lactifluusbrunnescens*	AV 05-083 (GENT)	Malawi	KR364019	De Crop et al. (2017)
*Lactifluuscaribaeus*	PAM/Mart 12-090 (LIP)	Martinique	KP691415	De Crop et al. (2017)
Lactifluuscf.castaneibadius	CL/MART06.019 (LIP)	Martinique	KP691417	De Crop et al. (2017)
*Lactifluuschiapanensis*	VMB 4374A (GENT)	Mexico	GU258297	Stubbe et al. (2010)
*Lactifluusclarkeae*	MN 2004002 (L)	Australia	KR364011	De Crop et al. (2017)
*Lactifluusflammans*	JD 941 (BR)	Congo	KR364078	De Crop et al. (2017)
*Lactifluusflocktonae*	JET1006 (MEL)	Australia	JX266621	Lebel et al. (2013)
*Lactifluusfoetens* **Type**	ADK 2840 (BR)	Benin	KR364023	De Crop et al. (2017)
*Lactifluusfoetens*	ADK 4411 (BR)	Togo	KX306937	De Crop et al. (2016)
*Lactifluusgymnocarpus*	EDC 12-047 (GENT)	Cameroon	KR364065	De Crop et al. (2017)
*Lactifluuslongivelutinus* **Type**	XHW 1565 (GENT)	China	KR364114	De Crop et al. (2017)
*Lactifluusluteolus*	AV 05-253 (GENT)	North America	KR364016	De Crop et al. (2017)
Lactifluuscf.murinipes	F.1890 (LIP)	Martinique	KP691418	De Crop et al. (2017)
Lactifluusaff. nebulosus	RC/Guad 11-023 (LIP)	Guadeloupe	KP691412	De Crop et al. (2017)
*Lactifluusnonpiscis* **Type**	BB 3171 (GENT)	Zambia	KR364030	De Crop et al. (2017)
*Lactifluusnonpiscis*	AV 11-137 (GENT)	Togo	KR364058	De Crop et al. (2017)
*Lactifluuspanuoides*	RC/Guy 10-024 (LIP)	French Guiana	KJ786647	De Crop et al. (2017)
*Lactifluus* aff. p*hlebonemus*	EDC 12-023 (GENT)	Cameroon	KR364062	De Crop et al. (2017)
Lactifluuscf.putidus	PAM/Mart 11-013 (LIP)	Martinique	KP691413	De Crop et al. (2017)
*Lactifluusrubrobrunnescens* **Type**	EH 7194 (GENT)	Indonesia	KR364115	De Crop et al. (2017)
*Lactifluus* sp.	RC/Guad 08-042 (LIP)	Guadeloupe	KP691414	De Crop et al. (2017)
*Lactifluus* sp.	G3185	French Guiana	KJ786694	De Crop et al. (2017)
*Lactifluuscaliendrifer* **Type**	KW 378 (GENT)	Thailand	MK517655	This study
*Lactifluuscaliendrife*r	KW 392 (GENT)	Thailand	KR364091	De Crop et al. (2017)
*Lactifluusrussilisporus*	RH 9674 (BRI, NY)	Australia	MK517654	This study
*Lactifluusrussilisporus* **Type**	RH 9398 (BRI, NY)	Australia	KR364097	De Crop et al. (2017)
*Lactifluus* sp.	PGK13-130	New Caledonia	KP691436	De Crop et al. (2017)
*Lactifluussubclarkeae*	RH 9231 (NY)	Australia	KR364095	De Crop et al. (2017)
Lactifluuscf.tanzanicus	AV 11-017 (GENT)	Tanzania	KR364053	De Crop et al. (2017)
*Lactifluustanzanicus* **Type**	TS 1277 (GENT)	Tanzania	KR364037	De Crop et al. (2017)
**Outgroup *Lactifluus***				
*Lactifluusacicularis*	KVP 08-002 (GENT)	Thailand	HQ318226	Van de Putte et al. (2010)
*Lactifluuscorrugis* s.l.	AV 05-392 (GENT)	USA	JQ753822	Van de Putte et al. (2016)
*Lactifluuscrocatus*	KVP 08-034 (GENT)	Thailand	HQ318243	Van de Putte et al. (2010)
*Lactifluusvitellinus*	KVP 08-024 (GENT)	Thailand	HQ318236	Van de Putte et al. (2010)
*Lactifluusvolemus*	KVP 11-002 (GENT)	Belgium	JQ753948	Van de Putte et al. (2016)

## Supplementary Material

XML Treatment for Lactifluus russulisporus

XML Treatment for Lactifluus caliendrifer
